# Consensus Statements on Airway Clearance Interventions in Intubated Critically Ill Patients—Protocol for a Delphi Study

**DOI:** 10.3390/life15081292

**Published:** 2025-08-14

**Authors:** Andrea A. Esmeijer, Prashant Nasa, George Ntoumenopoulos, Denise Battaglini, Deven Juneja, Lorenzo Ball, Stephan Ehrmann, Marcus J. Schultz, Frederique Paulus, Willemke Stilma

**Affiliations:** 1 Department of Intensive Care Medicine, Amsterdam University Medical Centres, 1105 AZ Amsterdam, The Netherlands; a.esmeijer1@amsterdamumc.nl (A.A.E.);; 2 Department of Anaesthesia and Critical Care Medicine, The Royal Wolverhampton Trust, New Cross Hospital, Wolverhampton WV10 0QP, UK; 3 Department of Physiotherapy, Royal Perth Hospital, Wellington Road, Perth, WA 6000, Australia; 4 Anesthesia and Intensive Care, IRCCS Ospedale Policlinico San Martino, 16131 Genoa, Italy; 5 Department of Surgical Sciences and Integrated Diagnostics (DISC), University of Genoa, 16132 Genoa, Italy; 6 Institute of Critical Care Medicine, Max Super Speciality Hospital, New Delhi 110017, India; 7 Médecine Intensive Réanimation, CIC INSERM 1415, CRICS-TriggerSep F-CRIN Research Network, CHRU Hospital Tours, 37044 Tours, France; 8 Centre d’étude des Pathologies Respiratoires, U1100, INSERM, Université de Tours, 37000 Tours, France; 9 Mahidol–Oxford Tropical Medicine Research Unit (MORU), Mahidol University, Bangkok 10400, Thailand; 10 Nuffield Department of Medicine, University of Oxford, Oxford OX3 7BN, UK; 11 Department of Anaesthesiology, General Intensive Care and Pain Medicine, Division of Cardiac Thoracic Vascular Anesthesia and Intensive Care Medicine, Medical University Vienna, 1090 Vienna, Austria; 12 Faculty of Health, Center of Expertise Urban Vitality, Amsterdam University of Applied Science, 1097 DZ Amsterdam, The Netherlands

**Keywords:** adult intensive & critical care, airway clearance, airway care interventions, humidification, nebulization, suctioning, mucus mobilization techniques, respiratory therapy, Delphi study

## Abstract

Intubated critically ill patients are susceptible to secretion accumulation because of compromised airway clearance. Various airway clearance interventions are employed to prevent complications arising from mucus retention. This Delphi study aims to collect global opinions in an international expert panel of ICU professionals on the usefulness of these various airway clearance interventions. A steering committee performed a literature search informing the formulation of statements. Statements are grouped into two distinct parts: (1) Humidification and Nebulization, and (2) Suctioning and Mucus mobilization techniques. For each part, a diverse panel of 30–40 experts will be selected, with concerted effort to involve experts from various medical specialties involved in airway clearance methods. Multiple choice questions (MCQs) or 7-point Likert-scale statements will be used in the iterative Delphi rounds to reach consensus on various airway clearance interventions. Rounds will continue until stability is achieved for all statements. Consensus will be deemed achieved when a choice in MCQs or a Likert-scale statement achieves ≥75% agreement or disagreement. Starting from the second round of the Delphi process, stability will be assessed using non-parametric χ^2^ tests or Kruskal–Wallis tests. Stability will be defined by a *p*-value of ≥0.05.

## 1. Introduction

Intubated critically ill patients often need airway clearance interventions due to the risk of secretion accumulation [1,2,3]. Frequently used airway clearance interventions are endotracheal suctioning [4,5] and subglottic drainage [6], active and passive humidification of inhaled air [7,8,9], nebulization of various agents to reduce the viscosity of mucus present in the airways [10], gravity-assisted drainage techniques [11], oscillatory techniques [12] and chest-wall compressions [13], and cough techniques [14,15,16]. This variety of airway clearance interventions are currently employed with inconsistent frequency to prevent complications arising from mucus retention.

The practice of airway clearance interventions varies widely with different criteria to initiate or cease the abovementioned interventions [17,18,19], and some of them are even used routinely, in all intubated critically ill patients without a clear clinical indication [17,18,19,20,21,22,23,24,25]. It should be noted that despite having potential benefits, some if not all interventions may have adverse effects [14,26] or may cause pain or discomfort [27,28], and some will increase medical waste [29]. There is a clear lack of high-quality recommendations regarding the use of airway clearance interventions. Available guidelines are mostly derived from clinical expertise in combination with low-level evidence [4,10]. At present, there is no international consensus concerning the practice of airway clearance interventions.

This protocol for an upcoming Delphi study aims to gather global opinions of ICU professional experts on the usefulness of airway clearance interventions in intubated critically ill patients. The document begins with a detailed description of the various airway clearance interventions and concludes by outlining the methods to be used in the planned Delphi study.

### 1.1. Airway Clearance Interventions

#### Endotracheal Suctioning

Suctioning is the most common method for clearing the airways of intubated critically ill patients and various techniques are employed [4,30]. Endotracheal suctioning can be performed using either an open or closed approach [30]. Open suctioning involves disconnecting the patient from the ventilator and using a single-use sterile catheter to remove mucus. In contrast, closed suctioning keeps the ventilator connected, minimizing the loss of airway pressures and reducing the risk of airway contamination. While open suctioning uses single-use suction catheters, closed suctioning catheters can be used for several days, which may reduce hospital waste.

Considerable uncertainty persists regarding the optimal approach to endotracheal suctioning and its impact on patient outcomes [31]. The debate between open and closed suctioning methods remains unresolved, with differing views on their respective benefits and risks. Additionally, there is ongoing discussion about the ideal frequency and best practices for minimizing potential complications associated with airway suctioning.

### 1.2. Subglottic Drainage

Subglottic drainage is employed to remove secretions that accumulate above the cuff of the endotracheal tube, an area that endotracheal suctioning cannot effectively reach [6]. This technique requires a specialized endotracheal tube with an additional lumen for continuous or intermittent suctioning. By removing these secretions, subglottic drainage prevents the aspiration of secretions into the lower airways. Even without these specialized endotracheal tubes, the removal of secretions through oropharyngeal suctioning before patient re-positioning may assist in preventing VAP [32].

However, questions persist about the broader applicability of the use of subglottic endotracheal tubes and their effectiveness. It is unclear whether these outcomes can be reliably replicated in diverse populations or across various clinical environments.

### 1.3. Humidification

Two methods are used to optimize inhaled air humidity: active humidification with a heated humidifier (HH) and passive humidification with a heat and moisture exchanger (HME) [7]. The HME filter retains heat and moisture during exhalation, passively humidifying inhaled air, while HH actively heats and moistens the air through a device in the ventilation circuit, resulting in higher humidity [8,9]. Currently, health professionals choose the humidification type based on clinical reasoning, with HH being costlier but preferred for prolonged ventilation [21,33].

Although both active and passive humidification methods are widely used, there is a lack of robust evidence guiding the choice between them. Additionally, the best humidification strategy for different patient populations remains a topic of debate, particularly concerning the prevention of complications like airway obstruction or infection.

### 1.4. Nebulization Therapy

Nebulized mucolytic agents are commonly used in ventilated patients to reduce mucus viscosity, whereas bronchodilators may aid clearance by widening small airways [10]. Nebulized agents are administered through a device that converts liquid medication into a fine mist or aerosol that can be inhaled directly into the lung [25].

The routine use of nebulization therapy of mucolytics and bronchodilators in invasively ventilated patients did not prevent accumulation of airway secretions in the endotracheal tube [26]. Moreover, the effects of nebulization therapy on clinical outcomes are still unclear. The decision to use these agents involves weighing their perceived benefits for mucus clearance against the risks of side effects, underscoring the importance of defining their role in the care of ventilated patients.

### 1.5. Gravity-Assisted Drainage Techniques

Gravity-assisted drainage can enhance mucus mobilization by promoting the cephalad movement of mucus towards larger airways, potentially improving the effectiveness of various airway clearance interventions. Prone positioning, recommended for patients with moderate to severe acute respiratory distress syndrome, may be particularly effective in promoting mucus movement towards larger airways and into the endotracheal tube, where it can be easily removed through suctioning [34].

The degree to which these interventions improve mucus mobilization and aid airway clearance, especially when used alongside other techniques, remains uncertain, as does their impact on clinical outcomes.

### 1.6. Oscillatory Techniques

High-frequency chest-wall oscillation (HFCWO) using an external vest rapidly compresses and relaxes the chest wall, which can enhance expiratory flow and improve mucus clearance by moving secretions cephalad [12]. HFCWO is predominantly used in non-intubated patients with hypersecretory conditions like cystic fibrosis and bronchiectasis, but it may also be beneficial in intubated critically ill patients.

The potential risks and clinical benefits of HFCWO in intubated patients, along with the subgroups most likely to benefit and the criteria for selecting, remain uncertain.

### 1.7. Chest-Wall Compressions

Chest-wall compressions, including vibration and percussion, are manual techniques used to mobilize secretions in invasively ventilated patients, moving them from peripheral to central airways [13]. These interventions are currently recommended for patients with a weak cough response to suctioning, and for patients with thick, tenacious airway secretions.

Evidence for the effectiveness of these interventions in invasively ventilated patients is limited. Questions remain about the optimal frequency, duration, and technique, as well as their impact on clinical outcomes.

### 1.8. Manual/Ventilator Hyperinflation and Mechanical Insufflation–Exsufflation

With manual hyperinflation (MH), the patient is temporarily disconnected from the ventilator, and a greater than normal tidal volume is manually inflated using a resuscitation bag with a slow inspiratory flow. This is followed by an inspiratory pause and a rapid release of the built-up airway pressure, which generates a high peak expiratory flow and aids in the movement of secretions. MH mimics a natural cough and helps prevent atelectasis [14].

The same technique can also be delivered through the mechanical ventilator, termed ventilator hyperinflation (VH), without the need for the disconnection of the patient from the ventilator and provides a potentially more accurate means to deliver the known pressure and/or tidal volume delivery during the intervention [15]. Mechanical insufflation–exsufflation (MI-E) devices, also known as cough-assist devices, provide a controlled artificial cough by delivering a predefined positive pressure followed by negative airway pressures. MI-E may be a more effective alternative to airway suctioning alone or MHI or VHI in certain situations as a means of mobilizing airway secretions [16], such as in patients with respiratory muscle weakness.

MH/VH are effective in improving mucus clearance over and above airway suctioning alone; however, their impact on clinical outcomes is uncertain, with concerns about risks like lung overdistension and hemodynamic instability. The use of MI–E in intubated patients is less studied than in non-invasive settings, leaving limited data on its safety and benefits. Potential risks, such as barotrauma, desaturation, intercranial hypertension, and hemodynamic instability, add to the uncertainty of its role in critically ill patients.

## 2. Materials and Methods

### 2.1. Design

This study will use the Delphi methodology to generate consensus on statements regarding the use of airway clearance interventions in invasively ventilated ICU patients [35,36,37]. A steering committee of 10 researchers, and clinicians with experience in airway clearance interventions (DB, LB, AE, SE, DJ, GN, FP, WS), including two Delphi methodologists (PN and MJS) was formed (see list of authors). The steering committee will perform the literature search for airway clearance interventions and formulate the statements based on the evidence. The members of the steering committee will identify and select an expert panel based on predefined selection criteria for the survey rounds. The steering committee will not participate as panellists in the survey rounds.

The study protocol is registered in clinicaltrials.gov (NCT06649734). This study will be conducted in accordance with principles of the ‘Declaration of Helsinki’ and reported following ACCORD guidelines [36]. The need for ethical approval was checked on 30th September 2024 at the NHS Health Research Authority of the Royal Wolverhampton NHS Trust, Wolverhampton, United Kingdom. According to the NHS health research authority, cross-sectional surveys involving healthcare professionals are exempt from formal ethics approval. Therefore, a formal IRB or ethics committee approval was not required prior to conducting this research. Participation in the study is voluntary, and completing and returning the survey or accepting the invitation for an interview, will be considered as explicit consent to take part in the study, a fact clearly stated in the initiation email. Panellists and their responses will be anonymous to each other but not to the coordinating members (AAE and PN) of the steering committee. Participants will be given the assurance that the confidentiality of their responses will be protected. Anonymous final group consensus will be available for review and publication.

### 2.2. Objectives

1. To review and structure existing evidence on the effectiveness of airway clearance interventions in intubated critically ill patients;
2. To collect global opinions on the usefulness of these various airway clearance interventions in invasively ventilated ICU patients.

This Delphi will be conducted in two separate parts. This division is necessary because the Delphi process consists of two distinct parts: (1) Humidification and Nebulization, and (2) Suctioning and Mucus mobilization techniques. The latter includes endotracheal and subglottic suction, gravity-assisted drainage techniques, oscillatory techniques, chest-wall compressions, and cough techniques. Each part focuses on different aspects of the topic and will be completed by mostly different panellists with specific expertise relevant to the respective areas. Presenting the results separately will allow for a more focused and clear discussion of the outcomes from each part, ensuring that the insights are tailored to the unique contributions of each group of experts. To ensure a representative selection of experts, a structured selection survey will be used to identify and invite professionals with the appropriate knowledge and experience in each domain. The selection survey will be used to determine whether experts contribute to both parts of the Delphi process or only one, depending on their specialization. The overall project timeline for both parts of the Delphi study is anticipated to be between 12 and 15 months.

### 2.3. Expert Panel

An international expert panel of at least 35 to 40 healthcare professionals representing ICU-nurses, respiratory therapists (or comparable versions, based on local implementation), physiotherapists, speech and language therapists, ICU-physicians, and pulmonologists with expertise in the field of invasively ventilated ICU-patients will be selected based on the following criteria:

○ At least 5 years of clinical experience, with care for invasively ventilated patients (teaching and non-teaching);
○ Participation in the development of a guideline or authorship of at least one paper on airway care for invasive ventilated patients in an international peer-reviewed journal;
○ No more than 70% of the panellists from each sex, and from each of the high-, low-, and middle-income countries;
○ Has a good command of the English language.

Purposive sampling will be utilized to recruit panellists by reviewing recent publications in the field of airway care for invasive ventilated patients. Selection of panellists will be guided by predefined criteria, with deliberate efforts to ensure a balance in gender and geographical representation. Panellists will receive an invitation in English via email detailing their role, the study’s objectives, and the Delphi process. Those who accept the invitation will participate in the Delphi rounds, with ongoing communication and regular progress updates provided to encourage their continued involvement. These updates will be provided in Delphi round reports after each round. To minimize dropout rates, at least three reminder emails will be sent during each round, and any feedback or concerns regarding the process will be addressed. Additionally, a schedule of Delphi rounds (each lasting 2 weeks) will be adhered to in order to maintain continuity.

### 2.4. The Delphi

#### 2.4.1. Step 1: Formulate List of Domains

In three consecutive meetings, the members of the steering committee discussed the airway clearance interventions to be included in this study. It was decided that the following interventions should be addressed:

○ Endotracheal suctioning and subglottic drainage;
○ Humidification;
○ Nebulization;
○ Mucus mobilization techniques.

Additionally, it was decided that the Delphi should provide information on the following:

○ Indications and contraindications;
○ Safety;
○ Effectiveness;
○ Efficiency;
○ Involvement of healthcare professionals.

#### 2.4.2. Step 2: Literature Search and Formulation of Statements

The steering committee conducted a literature review in the Medline database (PubMed) on airway clearance interventions in intubated critically ill patients, and selected studies if

○ One of the above airway clearance interventions was tested;
○ They were a randomized clinical trial;
○ They regarded safety, effectiveness, or efficiency;
○ They were published in or after the year 2000.

Studies were excluded if

○ They were performed in non-adults;
○ They were not a clinical study;
○ They did not address the outcomes of interest.

The PRISMA flow diagram will be used to depict the process of identifying, screening, and selecting studies for inclusion [38]. The available evidence, or the absence thereof, will guide the formulation of statements and questions for the domains included in the first Delphi round.

#### 2.4.3. Step 3: Preparation of the First Delphi Round

The panellists will receive a Delphi questionnaire via Google Forms, containing a series of questions related to the specified domains. Throughout the Delphi process, the identities and responses of the panellists will remain anonymous. Panellists will be asked to complete the questionnaire based on their knowledge and expertise in the subject area. The questionnaire will include multiple-choice questions (MCQs) and statements with a 7-point Likert scale. Responses and comments from panellists will be summarized into a report and provided as synthesized feedback in the following survey round.

#### 2.4.4. Step 4: Subsequent Delphi Rounds

The steering committee will evaluate the results of each round through online meetings. Statements will be revised, removed, or added as necessary, based on the feedback, comments, and responses received by the panel, The revised and remaining statements will be continued through subsequent rounds until a stable consensus or dissensus is reached (defined as ≥75% agreement or disagreement on a Likert scale and selected options in an MCQ). A summary of the results from each round will be shared with the panellists, and the survey process will be repeated using the updated questionnaire. The Delphi rounds will continue until the responses stabilize, with no more than 10% of panellists providing feedback that requires further adjustments. The process will be repeated until stability is achieved for all statements.

#### 2.4.5. Step 5: Final Consensus or Dissensus

Panel statements will be created based on the summary of the results of the last stable round. The panellists will be able to comment on the final survey results, consensus statements, and the article before publishing.

### 2.5. Patient Involvement

Patient perspectives will be incorporated by involving healthcare professionals who have personal experience as former critically ill ICU patients treated with invasive ventilation. Within the research network, we have a panel of such professionals, of whom three to five will be invited to participate in interviews on the results of the Delphi. By including the patient perspective, we aim to gain deeper insights into patients’ perceptions of airway clearance interventions and emotional or practical considerations that might only be visible through the lens of a patient’s perspective.

Results of the Delphi study will be presented to the patients and discussed during a qualitative interview. The interview will be guided by a topic list to ensure a structured discussion focussing on the patient perspective on all airway clearance interventions. The interview will be performed by two members of the steering committee (AE and WS) with expertise in qualitative research. The interviews will be transcribed verbatim and thematically analyzed by two researchers (AE and WS).

### 2.6. Analysis Plan

A descriptive analysis of each survey round will be performed. The stability of the responses will be analyzed from the second round onwards using the non-parametric Chi-square test or Kruskal–Wallis test. To define the stability of statements, a *p*-value of ≥0.05 will be interpreted as a lack of stability. Consensus will be defined when a choice in the MCQ or Likert-scale question has at least 75% of the votes from panellists. For the Likert-scale, the answering options are divided into disagreement (1 to 3), neutral (4), and agreement (5 to 7): 1 for strongly disagree, 2 for somewhat disagree, 3 for disagree, 4 for neutral, 5 for somewhat agree, 6 for agree, and 7 for strongly agree. Stability is assessed after two consecutive rounds of answers defined as consensus or dissensus. All data will be analyzed, regardless of the completeness of answers.

The steering committee will compile the results from each Delphi round and share them with the panellists, together with the survey of the following round. These results will demonstrate the degree of consensus or dissensus achieved for each statement. In the final results, percentages of voting for each answering option will be reported.

The findings from this study will be presented in two separate reports: (1) Humidification and Nebulization, and (2) Suctioning and Mucus mobilization techniques.

### 2.7. Ethics and Dissemination

The study will be conducted in strict accordance with the principles of the Declaration of Helsinki and reported following ACCORD guidelines. An ethical approval waiver was granted, due to the nature of this research. The main ethical considerations of this study involve group biases and peer pressure. Therefore, the panellists will remain anonymous to each other throughout the Delphi process. Participation in the study is voluntary, and completing and returning the survey will be considered as explicit consent to take part in the study. Patients’ representatives will be involved to ensure their perspectives are considered throughout the research process. The study results will be published in a peer-reviewed journal with the authorship agreed as per ICMJE requirements.

### 2.8. Study Progress to Date

The formulation of the statements was started by the Steering Committee. The first round of the Delphi process will be executed shortly thereafter.

## 3. Discussion

This Delphi study aims to collect global opinions on the usefulness of airway clearance interventions in intubated critically ill patients, leveraging the collective expertise of an international panel of healthcare professionals. The study focuses on identifying key criteria for the indications and contra-indications for these interventions, and identifying perceived outcomes related to these interventions. Through consensus building, we seek to clarify areas of agreement and disagreement among international experts, providing a structured foundation to guide clinical decision making and prioritize future research. Ultimately, this aims to improve patient care and reduce the unnecessary use of potentially harmful and costly interventions.

Managing airway clearance in intubated, critically ill patients is challenging due to the lack of high-quality evidence and guidelines. The variability in practice can expose patients to interventions that may be effective, potentially harmful, or unnecessarily uncomfortable [21]. The urgent need for evidence-based guidance in this vulnerable population underscores the value of consensus methods like the Delphi technique, which systematically gathers expert opinions to inform clinical practice where evidence is limited or contradictory. While the Delphi methodology is sometimes viewed as less rigorous than randomized controlled trials, it remains a pragmatic and reliable tool for generating expert-driven consensus [39].

To ensure the credibility of this Delphi study, we will adhere to established standards: thoroughly reviewing existing evidence, employing predefined criteria for expert selection, maintaining anonymity in responses, and clearly defining the procedures for achieving consensus [40,41]. Our methodology, including the selection process, response rates, and any limitations, will be transparently reported. By following these rigorous standards, this Delphi study aims to produce credible, consensus-based recommendations for the safe and effective use of airway clearance interventions in intubated, critically ill patients, paving the way for more consistent and evidence-based clinical practice.

Our Delphi study has strengths. The Delphi study will be groundbreaking in its evaluation of airway clearance interventions across a wide range of techniques specifically in intubated, critically ill patients. By following a rigorous and methodologically robust Delphi, our study will provide clarity and guidance on the best practices in this vulnerable group of patients. We aim to recruit a diverse panel of experts from various geographical regions, representing both resource-rich and resource-limited settings to ensure a broad range of perspectives. This panel will consist of a multidisciplinary group of professionals, including ICU nurses, respiratory therapists, physiotherapists, intensivists and pulmonologists, and other healthcare professionals in equivalent roles based on local practices as long as they have expertise in the care of invasively ventilated ICU patients. In addition, we will ensure gender and age balance within the panel to provide a wide representation of views and experiences. By including this diverse range of professionals, our study will capture broader insights into regional practices and resource availability, making the results widely applicable. Selection of the panellists will be based on predefined criteria and a self-assessment survey to ensure the inclusion of individuals with relevant experience and knowledge in airway clearance interventions. To avoid the influence of peer or group bias, expert responses will remain anonymous throughout the Delphi rounds, allowing participants to provide their opinions independently and without peer pressure. The final consensus will emerge through several iterative rounds, with the endpoint being reached when responses show stability across all statements.

This study will also have limitations. One potential challenge is that the interpretation of statements may influence panellists’ responses. However, this will be mitigated by incorporating open feedback from panellists in each round and assessing the consistency of responses over multiple iterations. Another limitation could arise from differences in local practices or access to certain airway clearance techniques, which may affect responses. To address this, we will limit the panellists from any single geographical region to no more than approximately one-third, ensuring a well-balanced global perspective and providing the published literature on the different techniques.

## 4. Conclusions

An international panel of experienced healthcare professionals will contribute their collective insights through this Delphi study on the effectiveness and safety of various airway clearance interventions in intubated, critically ill patients. By identifying key criteria for their use, the study aims to offer consensus-based recommendations, ultimately to improve clinical practice and outcomes of invasively ventilated patients in critical care units worldwide.

## Data Availability

The original contributions presented in this study are included in the article. Further inquiries can be directed to the corresponding author.
